# Osteosarcoma of the jaws: A case report

**DOI:** 10.1016/j.ijscr.2022.106909

**Published:** 2022-03-04

**Authors:** Amminou Loubna, Taleb Bouchra

**Affiliations:** a Department of Oral Surgery, Dental Center of Treatment and Diagnosis (Ibn Sina Hospital), Rabat, Morocco; b Faculty of Dentistry of Rabat, Mohammed V University, Morocco

**Keywords:** Osteosarcoma, Jaws, cancer

## Abstract

**Introduction:**

Osteosarcomas of the jaws are very rare accounting for only 6 to 7% of all osteosarcomas and 1% of all head and neck malignancies.

The clinicopathological characteristics as well as the radiological characteristics are not specific, which represents a diagnostic dilemma. Indeed, this lesion is often mistaken for benign lesions of the maxillary.

**Case presentation:**

We report a case of osteosarcoma of the jaws in a 45 year man who consulted in our service for a simple painless oral swelling evolving since a five months.

The lesion was excised and histopathological report confirmed the diagnosis of osteosarcoma of the jaw.

**Discussion:**

Osteosarcomas are a rarely progressive tumor in the oral cavity that can show up as a case of a benign process of dental origin. During our practice, we often meet similar cases which are treated by usual medication, while a whole malignant process is in the progress and can be life-threatening. The case we present illustrates this perfectly, and owing to a deep clinical investment, the malignant process was stopped at its start.

**Conclusion:**

The objective of presenting this case is to draw the attention of the doctors that any lesions even of benign appearance can hide a malignant process. Consider this point and integrate it into its diagnostic approach should be undertaken and should lead to a deepening of the clinical examination by comparing the clinical, radiological and histological data.

## Highlights

• Never underestimate a lesion of usual appearence; a whole malignant process can be hidden.
• A dippening of clinical and paraclinical investigations is an approch to adopt.
• Early diagnosis can improve the management of the desease and save lives.

## 1 Introduction

Osteosarcoma, is a rare malignant bone tumor arising from primitive bone forming mesenchyme, most often arises in the metaphyses of long bones of the extremities [1]. Its location in the jaws is extremely rare [2].

Dentists are the first to evaluate the lesion in 45% of cases. The diagnosis is often wrong leading to treatment of the lesion in 2/3 of cases with tooth extraction and half with antibiotics [3].

Swellings are the most common presenting symptoms in cases with osteosarcoma. Pain, paresthesia, and ulcerations are less common [4].

The radiological examination is not specific; it can show a lytic, sclerotic or mixed image [4].

This article has been reported in line with the SCARE criteria [5].

## 2 Case presentation

A 45-year-old man in good general health condition, without any known health problem or any genetic pathology detected, consulted at the university center for dental treatment and care at the faculty of dental medicine at Rabat, for a maxillary swelling evolving for five months.

During the interview, the patient reports the absence of pain or paraesthesia. Exobuccal examination doesn't report any abnormalities. No lymphadenopathy was palpable.

**Fig. 1:**
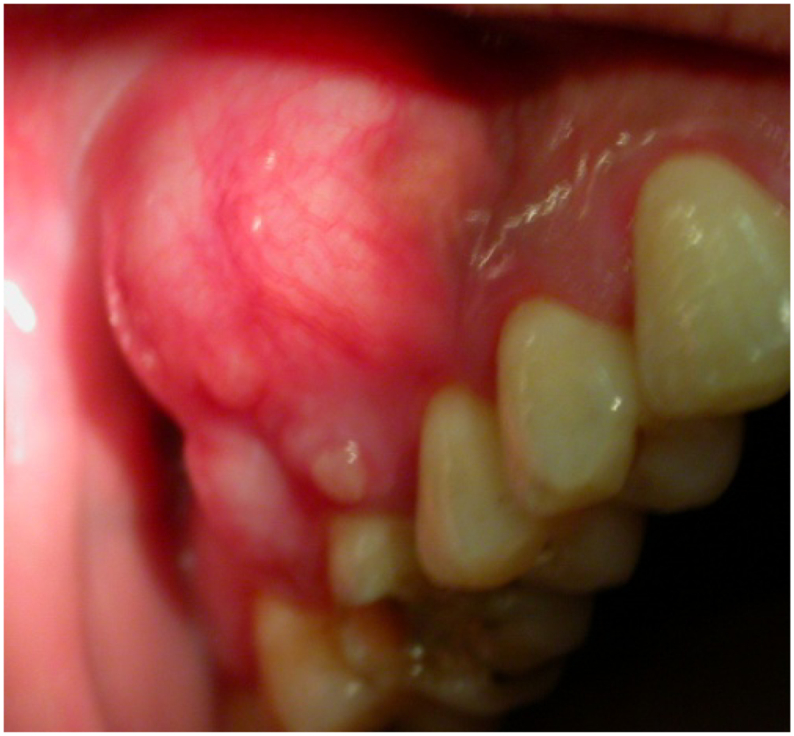
Endobuccal view showing the mass in front of the first Molar located on the right side of lips.

The endobuccal inspection shows the presence of a round swelling located at the level of the mucosa of the right upper molar-premolar region (Fig. 1). The swelling is 2 cm in diameter covered with normal mucosa. On palpation, the mass is firm. No fluctuation or crackling is noted. The vitality test of the teeth is positive. No mobility of those teeth is noted.

**Fig. 2:**
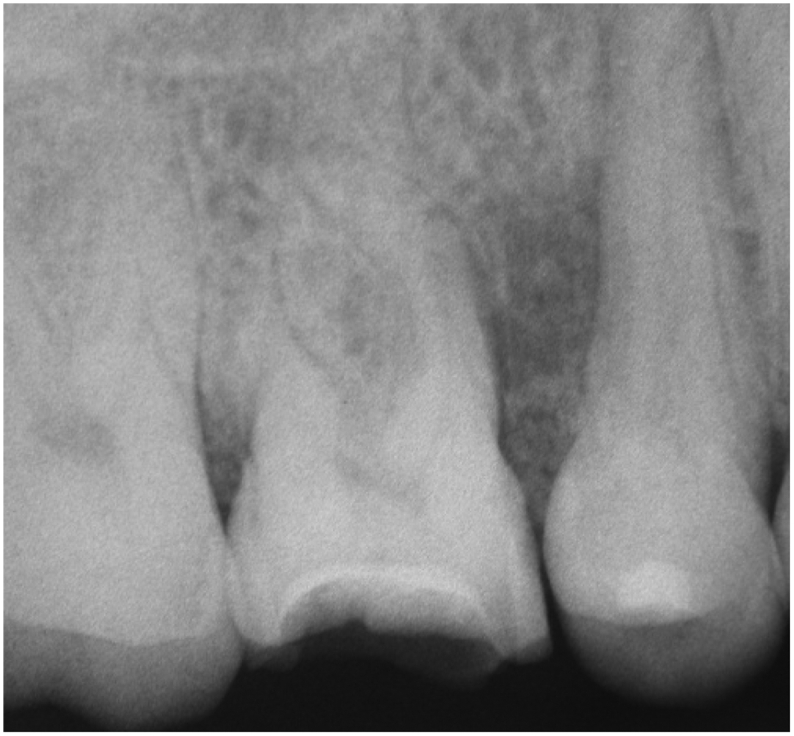
Pré-operative radiography showing the radiolucency between the first molar and second premolar.

Radiographic exam shows a radiolucency located on the middle third of the roots of the first right molar and the second right premolar (Fig. 2).

**Fig. 3:**
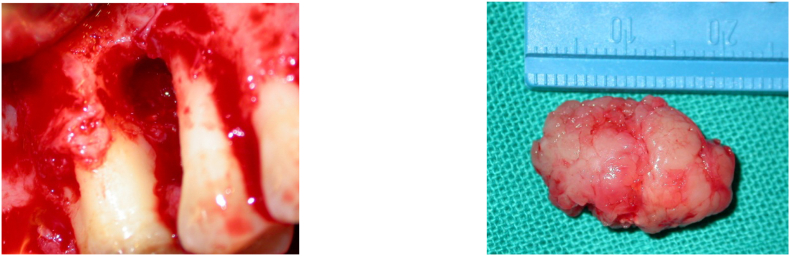
Excision of the lesion and bone curettage.

**Fig. 4:**
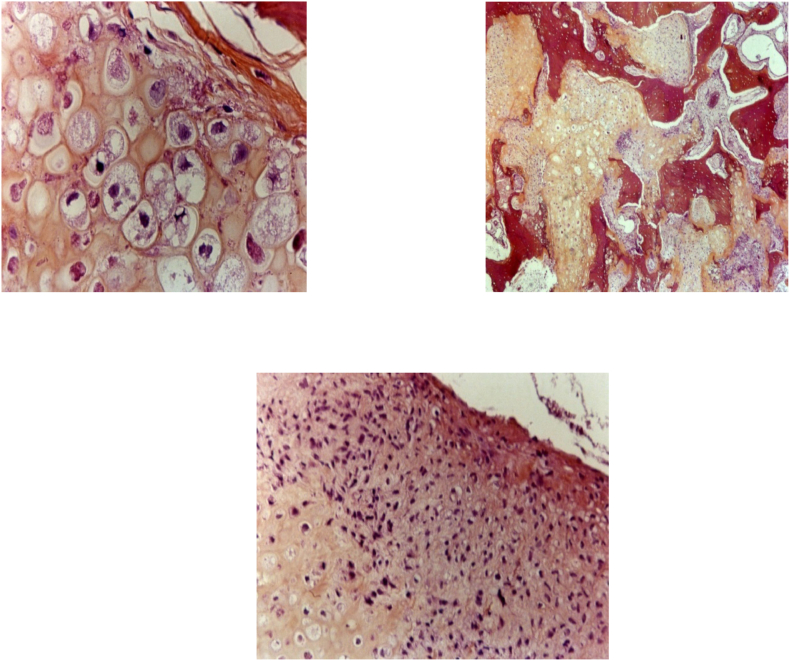
Histopathological appearance of osteosarcoma.

An excisional biopsy was performed at the center for dental treatment (Fig. 3) and an anatomopathological examination was carried out confirming the diagnosis of osteosarcoma (Fig. 4).

The patient was transferred to a specialized unit where an extension assessment was performed.

There was no extension beyond the oral site and a wide local excision with tooth extraction was performed. No other treatments were performed.

The patient benefited from a follow-up at the rate of three times the first year then one time each year. No malignant process or metastasis has been identified.

## 3 Discussion

Osteosarcoma of the jaws accounts only for 6% to 7% of all osteosarcomas [6], [3]. It represents approximately 0.2% of all malignancies [7]. Often found in long bones, its occurrence in the jaws remains uncommon [2].

The patients are usually older than those who suffer long bone sarcomas, with a rare incidence of metastasis [2], [4]. More men than women are affected [2].

The mandible is marginally more commun site than the maxilla and tends to occur in the body and the ramus, whereas the canine and premolar are more likely site in the maxilla [6], [7].

The etiological mechanisms are unknown [2]. Environmental factors such as ionizing radiation and chromium oxide, a radioactive scanning agent, have also been incriminated [2], [8], [12].

The most common presenting symptoms are swelling, pain [3], [2]. Periapical inflammation and loosening of teeth are also found [2]. Other symptoms like hypothesia and paraesthesia of involved nerves, eye symptoms, trismus, nasal obstruction, epistaxis and gingival inflammation can be seen as well as numbness of the lower lips [2].

In view of these non-specific symptoms, the osteosarcoma is poorly diagnosed as a periapical lesion or odontogenic lesion [9].

Other differential diagnoses include fibrous dysplasia, ossifying fibroma, chondromyxoid fibroma [1].

Radiographic examination usually shows a lytic, sclerotic or mixed lesion with soft tissue extension in the majority of cases [2], [4].

If the tumor invades the periosteum, many thin irregular spicules of new bone may develop outward and perpendicular to the surface of the lesion producing the so-called “sun ray appearance” [4], [8]. However, this image cannot be considered pathognomonic of osteosarcoma [10].

Periodontal ligament space widening and lamina dura attenuation around a tumor are other features that may present in jaw osteosarcomas [2], [4].

The importance of special investigations such as computerized tomography and magnetic resonance imaging lies in assessing the size of the lesion for staging, intramedullary and extramedullary involvement, tumor calcification and invasion into adjacent tissues particularly pterygopalatine fossa, infra temporal fossa and cranial cavity [6], [8].

Histologically, the neoplastic cells are predominately spindle-shaped, with minimal atypia and relatively few mitoses [1].

A variable osteoid production is described with several subtypes. Among these, we find osteoblastic, chondroblastic, or fibroblastic subtypes [1], [2], [8].

The two therapeutic modalities used in the primary treatment of osteosarcoma include chemotherapy and radical surgery.

Chemotherapy is indicated for head and neck osteosarcomas that are unresectable, with metastases, in unusual locations (base of skull) or with aggressive histopathology [11].

Radical surgery in the mandible consists of hemi-mandibulectomy. Maxillary lesions are often difficult to be treated as involvement of maxillary sinus, pterygopalatine fossa and orbital fossa often masks the tumor until extensive spread. Often, maxillectomy is inevitable. If cervical lymph nodes are involved, neck dissection would improve the survival [8].

Osteosarcoma is particularly resistant to radiotherapy, which should only be considered to prevent local recurrence owing to the possibility of tumor-positive margins after surgical resection [8], [11].

Osteosarcoma of the jaws have better prognosis than conventional osteosarcomas [7]. Regional metastasis is rare [8], [11].

## 4 Conclusion

Osteosarcoma of the jaws is a rare malignant lesion. However, dentists are most confronted with clinical and radiological signs.

Hence, this article's main objective is to show the importance of early diagnosis.

The latter is the key to a better prognosis avoiding the extension of the lesion and optimizing its management.

## Source of funding

No external funding.

## Ethical approval

Not required.

## Consent

Written informed consent was obtained from the patient for publication of this case report and accompanying images. A copy of the written consent is available for review by the Editor-in-Chief of this journal on request.

## Author contribution

Dr Amminou Loubna: Drafting the work conception and design of the work.

Dr Taleb Bouchra: Revising the work critically, Acquisition and analysis of work, Final approval to the version to be published.

## Guarantor

Amminou Loubna.

## Provenance and peer review

Not commissioned, externally peer-reviewed.

## Declaration of competing interest

The authors declare no conflict of interest.
